# Peri-Trapezium Peri-Trapezoid Trans-scaphoid Open Fracture Dislocation of the Carpus: A Case Report

**DOI:** 10.7759/cureus.62891

**Published:** 2024-06-22

**Authors:** Hasan Aleisawi, Khalid Alosaimi, Hussain Algawahmed

**Affiliations:** 1 Orthopedic Surgery, King Faisal Specialist Hospital and Research Centre (KFSHRC), Riyadh, SAU; 2 Orthopedic Surgery, King Saud Medical City, Riyadh, SAU; 3 Orthopedics, Hand, Elbow, and Shoulder Surgery, Johns Hopkins Aramco Healthcare, Dhahran, SAU

**Keywords:** case report, axial carpal fracture dislocation, scaphoid, carpal dislocation, longitudinal disruption

## Abstract

The axial fracture dislocation of the carpal bones is a poorly understood injury. These injuries are often associated with nerve injuries, soft tissue injuries, and amputations. An optimal treatment is not yet known. We present a rare case of an open trans-scaphoid axial fracture dislocation of the carpus, characterized by a unique mechanism of injury. The patient received timely treatment, including irrigation and debridement, as well as reduction and fixation of the carpal fracture dislocation. It is essential to understand that these injuries tend to result in poor clinical outcomes, even with optimal treatment. Therefore, engaging in meaningful discussions and setting realistic expectations with patients is imperative.

## Introduction

The longitudinal disruption of the carpal arch, a rare and clinically significant event, was initially documented by Oberts in 1901 [1]. These injuries, which are infrequent and typically result from severe trauma, are prone to being overlooked in clinical assessments. Garcia-Elias and colleagues have classified these injuries into three distinct categories: axial-radial, axial-ulnar, and combined injuries [2]. Notably, the conjunction of a scaphoid fracture with axial disruption of the carpus represents an exceedingly rare clinical presentation, one that is not encompassed within the classification proposed by Garcia-Elias et al. [2]. In this context, we report a singular case of longitudinal disruption of the carpus concomitant with an open scaphoid fracture. This case study encompasses a detailed discussion of the therapeutic interventions employed and the subsequent clinical outcomes.

## Case presentation

A 37-year-old right-handed male was admitted to the emergency department (ED) following an incident involving a direct impact from a forklift truck on his left hand. The patient, while standing with his hand resting on a wall in full wrist extension, experienced an accidental hit to the dorsum of his hand from the forklift, operated by a co-worker. Subsequently, the materials loaded on the forklift fell onto the patient's hand. He reported severe pain post injury, accompanied by diminished movement in all fingers and the wrist. Upon initial clinical examination, the patient was found to be hemodynamically stable with an isolated hand injury. Local examination revealed a wound on the dorsoradial aspect of the wrist, significant swelling, restricted range of motion (ROM) in the radial three fingers and the wrist, along with marked tenderness over the snuffbox area. The capillary refill time was less than two seconds, and sensory examination showed intact responses for the ulnar, median, and radial nerves.

**Figure 1:**
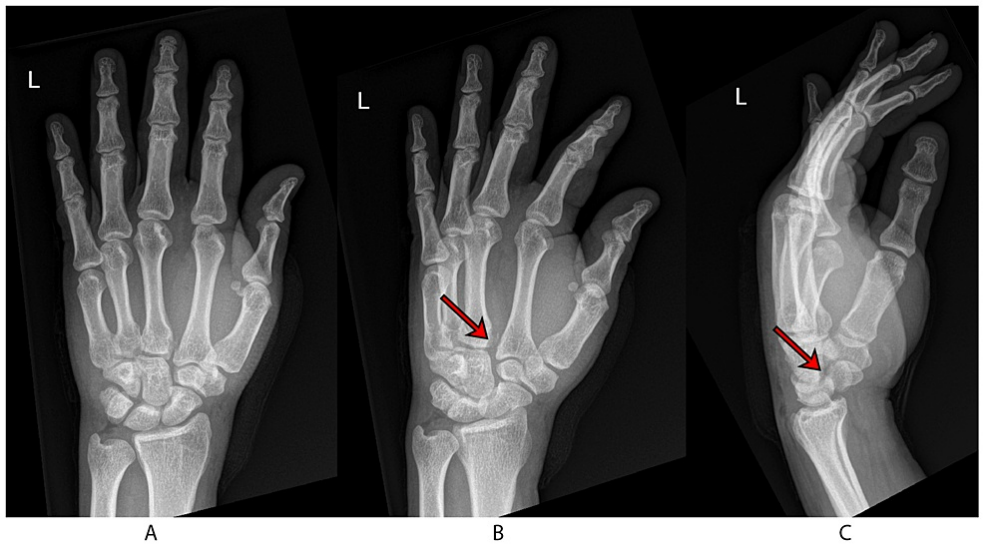
Preoperative images A: posteroanterior radiograph of the wrist; B: the oblique wrist view shows the presence of diastasis, as indicated by an arrow; C: the lateral wrist view shows a displaced scaphoid fracture, as indicated by an arrow.

**Figure 2:**
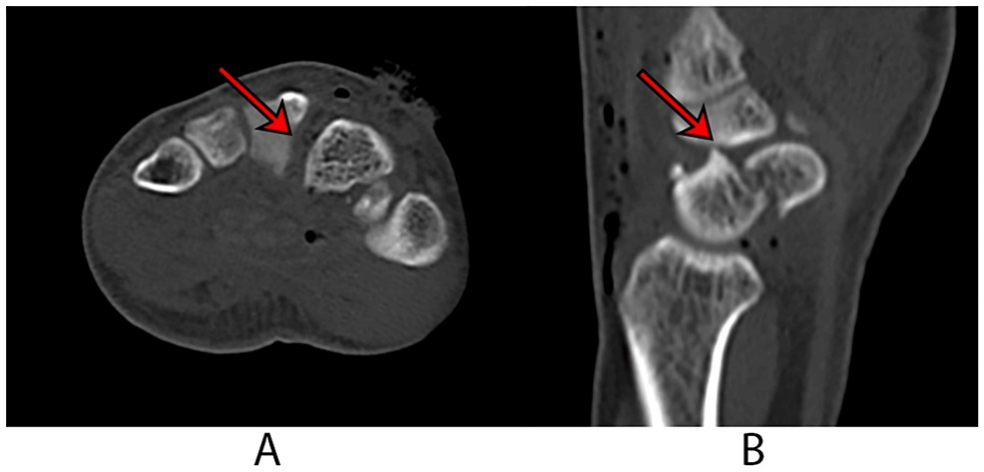
Computed tomography scan A: An axial view of the CT scan shows diastasis of the second and third metacarpal bases, indicated by an arrow; B: A sagittal reconstruction view of the CT scan shows the scaphoid fracture, as indicated by an arrow.

Radiographic evaluation of the hand and wrist revealed a displaced fracture of the left scaphoid, as well as a widening between the bases of the second and third metacarpal bones and the capito-trapezoidal joint (Figure 1). A computed tomography (CT) scan further identified a displaced fracture at the waist of the scaphoid, diastasis between the bases of the second and third metacarpal bones, and the capito-trapezoidal joint (Figure 2). Standard open fracture management was initiated, and a short arm backslab was applied.

**Figure 3:**
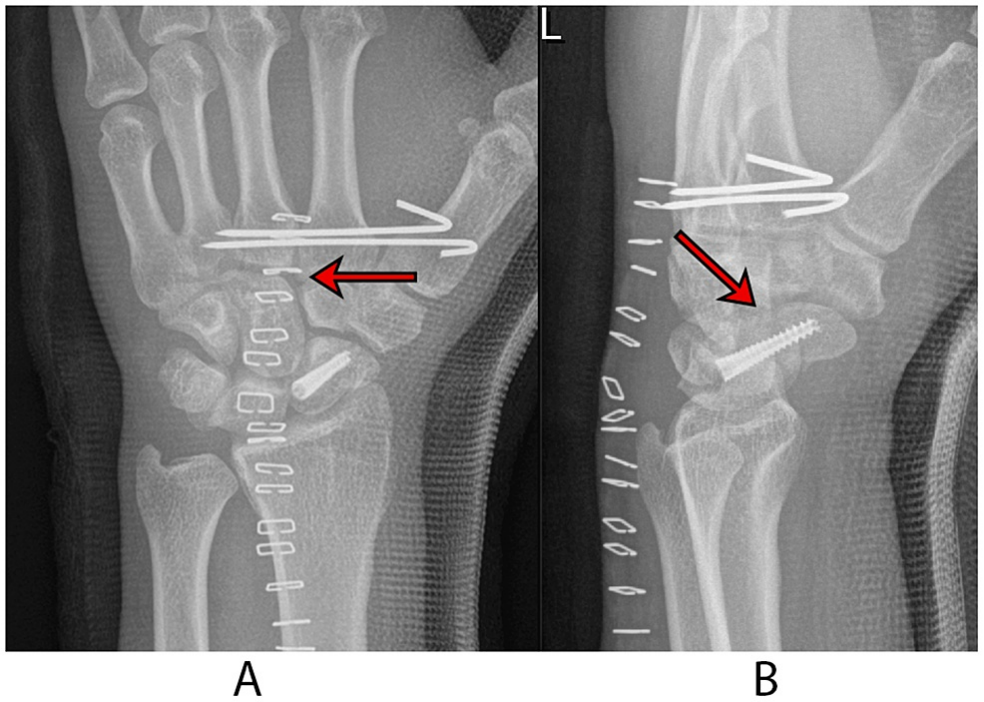
Postoperative images A: the posteroanterior view of the wrist shows two Kirschner wires transfixing the second and third metacarpal bones with excellent reduction of diastasis, as indicated by an arrow; B: the lateral view of the wrist shows excellent reduction and fixation of the scaphoid fracture, as indicated by an arrow.

Given the complexity of the open scaphoid fracture coupled with the longitudinal disruption, a surgical intervention was deemed necessary. The patient was taken to the operating room urgently. He was positioned supine with an arm board, and a tourniquet was applied to the proximal upper arm. The wound, extending obliquely for 3 cm between the bases of the first and second metacarpal bones, was found communicating with the carpal bones. Standard irrigation and debridement (I/D) were performed, resulting in a healthy, closable wound. The scaphoid fracture's significant displacement necessitated a dorsal approach to the wrist, as it was not accessible through the traumatic wound. A Z-plasty incision of the extensor retinaculum and an inverted T-capsulotomy of the wrist joint were performed, ensuring tendon protection. The scaphoid fracture was exposed, meticulously cleaned, and irrigated. Fracture reduction was achieved using Kirschner wires (K-wires) as joysticks, confirmed under image guidance, and secured with preliminary K-wire fixation followed by headless screw fixation in a standard fashion. The diastasis at the capito-trapezoidal joint and the second and third metacarpal bases improved after the scaphoid reduction and fixation. Closed reduction of the diastasis was achieved using a percutaneously placed pointed reduction clamp, followed by K-wire fixation from the radial side of the second to the third metacarpal base. Layered closure was performed, and the hand was immobilized in a thumb-spica cast with a sling. The patient's postoperative course was uneventful, with normal distal neurovascular function and satisfactory radiographic results (Figure 3).

**Figure 4:**
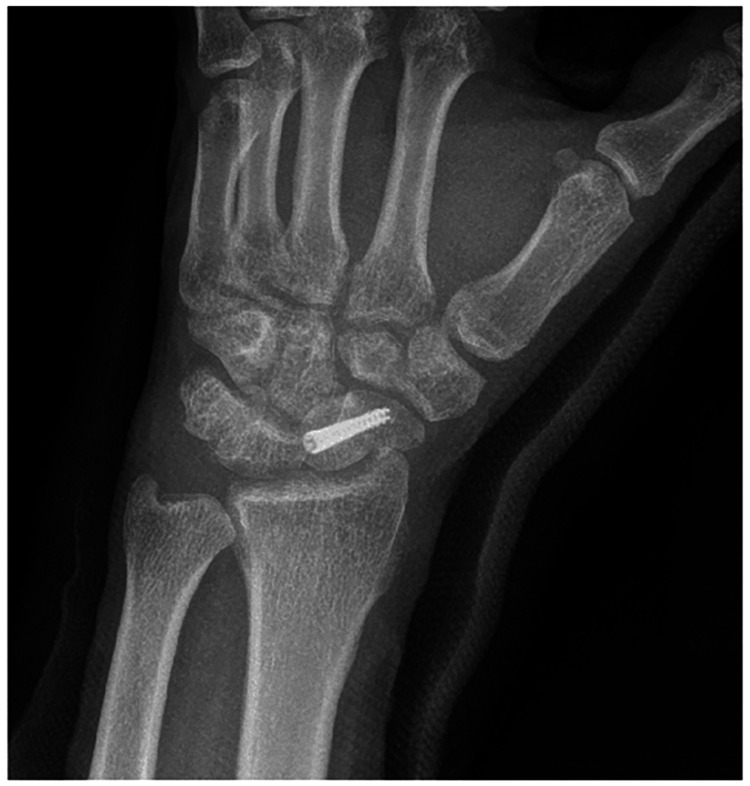
Radiographic image obtained during the seven-week follow-up An oblique view of the wrist shows acceptable alignment.

**Figure 5:**
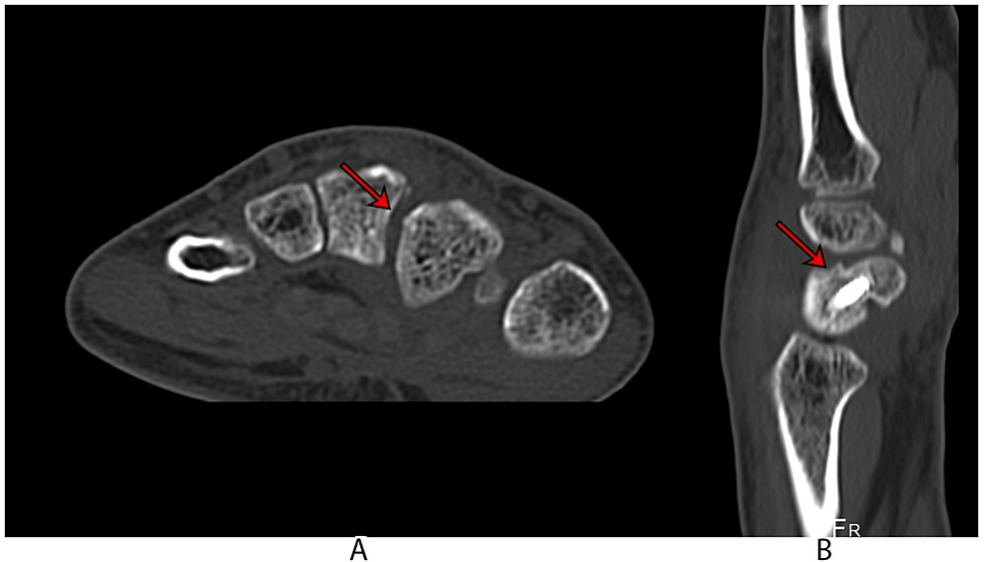
Postoperative CT scan images of the wrist A: The axial view of the CT scan shows persistent mild diastasis of the second and third metacarpal bases, as indicated by an arrow; B: The sagittal reconstruction view of the CT scan shows a united scaphoid fracture, as indicated by an arrow.

At the two-week follow-up, the wound had healed, and staples were removed. The patient reported no symptoms. At seven weeks, the K-wires and the cast were removed, and occupational therapy was initiated (Figure 4). At three months, the patient reported no discomfort, exhibiting full extension in the fingers, though flexion at the second and third metacarpophalangeal (MCP) joints was limited to 60 degrees. Grip strength was at 20% of the normal side. The patient was encouraged to adhere to occupational therapy for optimal recovery. By the six-month mark, he had demonstrated ongoing improvement. Computed tomography imaging confirmed over 50% union of the scaphoid, with no signs of avascular necrosis or early arthritic changes. The fractures at the bases of the second and third intercarpal joints were fully healed, with a tiny bony fragment observed in the inter-metacarpal joints (Figure 5). Grip strength and ROM were significantly improved.

**Figure 6:**
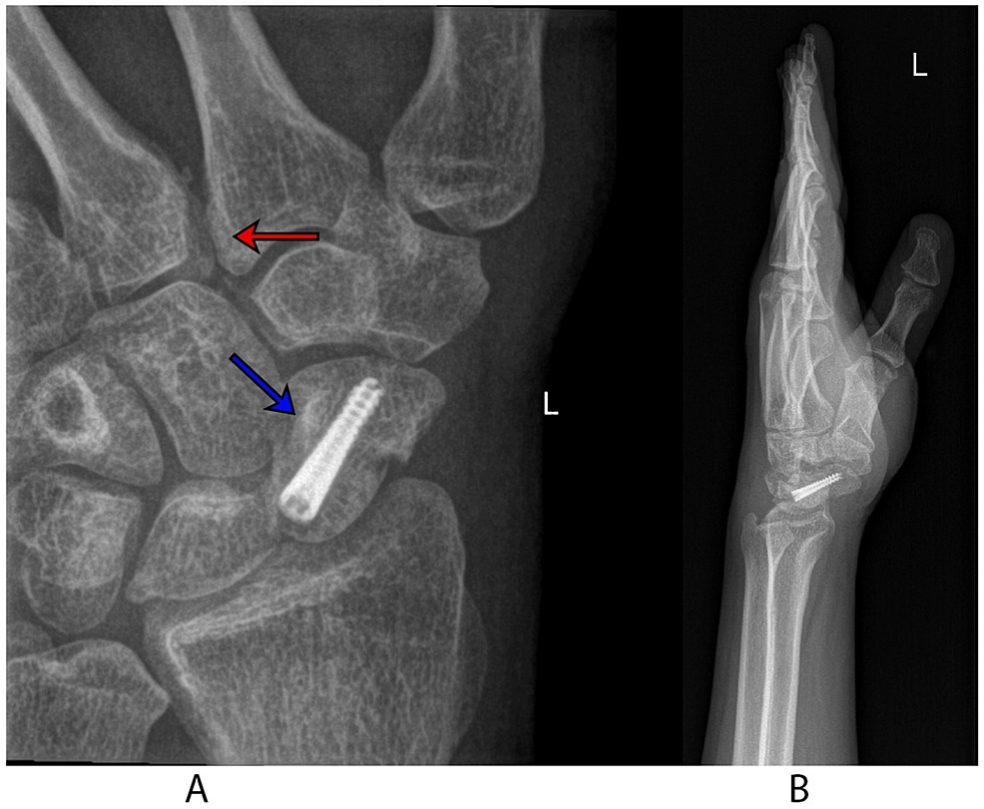
Final follow-up radiographs A: the scaphoid view demonstrates healing of the scaphoid fracture (blue arrow) as well as acceptable reduction of the diastasis (red arrow); B: the lateral view demonstrates a good radiographic outcome.

The final one-year follow-up showed the patient to be symptom-free. The ROM, as measured by a goniometer, indicated wrist extension and flexion at 50° on the injured side versus 60° and 65° on the normal side, respectively. Digital, radial-ulnar deviation, and supination-pronation movements were full. Handgrip strength, assessed three times on both sides using a dynamometer, was 65% of the normal side. The Disabilities of the Arm, Shoulder, and Hand (DASH) score was 10/100. Radiographs confirmed bony union without signs of arthritic changes, deformity, or instability (Figure 6).

## Discussion

This case presents with unusual and complex features, notably the presence of a peri-trapezium peri-trapezoid trans-scaphoid axial fracture dislocation of the carpal bones. Garcia-Elias et al. have provided a classification for axial dislocations of the carpal bones, encompassing axial-radial injury, axial-ulnar injury, and combined column injury [2]. This injury is also termed longitudinal dissociation of the carpal bones because the axis of dissociation of the carpal bones is in line with the axis of the forearm. The presented case would be classified as an axial-radial fracture-dislocation.

Regrettably, axial carpal dislocations are often severe and associated with neurovascular injuries and complications, leading to poor clinical outcomes [2, 3]. Shannon et al., in a review of 3485 patients treated for carpal dislocations, fractures, and/or metacarpal fractures over 25 years, identified only 37 patients with axial dislocations of the carpus, constituting 1.1% of cases [4].

The Garcia-Elias classification, while comprehensive, does not account for proximal row injuries, focusing instead on the distal row. Recent literature, however, includes reports of various injuries to the proximal row [3, 5, 6]. Since Oberts' first report in 1901, axial carpal fracture dislocations have typically been documented in case reports and small series. The occurrence of a trans-scaphoid fracture in conjunction with axial carpal dislocation is exceptionally rare. Our case is unique, involving only the radial column, contrasting with previous reports involving the ulnar column [3, 5, 6]. This pattern of injury could expand the Garcia-Elias classification to include ‘trans-scaphoid’ fractures in proximal row injuries.

Garcia-Elias suggested that the injury results from a dorsal-to-volar compressive force causing carpal bone splitting. Conversely, Shannon et al. proposed that these injuries result from a true axial force, transmitted from proximal to distal, rather than an axial crush injury [4]. The mechanism of injury is thus complex and extends beyond a simple axial force.

Given the rarity of such injuries, clear treatment guidelines are lacking. General principles of fracture and dislocation reduction and fixation apply. In this case, reduction of the diastasis was initially achieved through the traumatic wound, followed by pinning using K-wires. However, postoperative CT revealed diastasis between the second and third metacarpal bone bases, the clinical significance of which remains unclear. Some authors speculate that diastasis might contribute to handgrip weakness, while others suggest that handgrip weakness is a result of flattening of the hand arch [6, 7]. Nonetheless, we advocate for an open reduction approach in axial carpal dislocations to mitigate such complications.

Despite achieving a near-full, painless ROM in the digits and wrist, the patient's grip strength was markedly decreased. This finding aligns with reports from several authors [2, 7]. It is important to consider potential differences in handgrip strength between dominant and non-dominant hands [8]. Shannon et al., in their retrospective study, the largest to date, of axial-radial and axial-ulnar injuries, reported poorer outcomes in axial-radial injuries, with an average Mayo Wrist score of 52.2 (less than 60 is considered poor) and no patients achieving a good outcome [4]. Additionally, Garcia-Elias et al. identified nerve injury as a significant prognostic factor, with most patients having ulnar or median nerve injuries showing poor outcomes [2].

## Conclusions

Trans-scaphoid axial carpal fracture dislocation constitutes a rare and complex injury, often prognosticated with a guarded clinical outcome. Patients with axial-radial carpal fracture dislocations are generally anticipated to experience suboptimal outcomes, manifesting notably in reduced handgrip strength. These injuries are frequently accompanied by significant soft tissue and neurovascular complications, necessitating a high degree of clinical vigilance.

The treatment for these injuries should encompass meticulous reduction of the dislocated joints, stabilization, and comprehensive management of associated soft tissue envelope injuries. The intricacies of these injuries must not be underestimated, given their potential for long-term morbidity. To facilitate optimal recovery, early and targeted rehabilitation is imperative, focusing on the restoration of handgrip strength and ROM. 
